# A Fuel Cell Power Supply System Equipped with Artificial Gill Membranes for Underwater Applications

**DOI:** 10.1002/advs.202410358

**Published:** 2025-01-10

**Authors:** Lucas Merckelbach, Prokopios Georgopanos

**Affiliations:** ^1^ Helmholtz‐Zentrum Hereon Institute of Coastal Ocean Dynamics Max Planck Str. 1 21502 Geesthacht Germany; ^2^ Helmholtz‐Zentrum Hereon Institute of Membrane Research Max Planck Str. 1 21502 Geesthacht Germany

**Keywords:** artificial gills, autonomous underwater vehicles, fuel cells, ocean gliders, underwater sensors, polymer membranes

## Abstract

This work proposes a fuel cell power supply system for underwater applications (e.g., autonomous underwater vehicles), where artificial gills, based on a polymer membrane, harvest the required oxygen from the ambient water. In this system, a circulating air‐flow continuously supplies a proton exchange membrane fuel cell with oxygen, which is replenished using a polymer membrane. The membrane serves as the interface between the circulating air‐flow and the ambient water, preventing water flux while allowing an oxygen flux across the membrane, driven by a partial oxygen pressure gradient. To demonstrate the feasibility of this concept, a prototype system was built based on guidelines derived from a mathematical model that was developed to describe the oxygen transfer process. A computational fluid dynamics model is developed and validated against the measurements from the prototype, resulting in a digital twin. The analysis indicates that the proposed power supply system has the potential to be superior to any battery‐based solution currently available.

## Graphical abstract

Aprototype power supply based on a hydrogen fuel cell and an artificial gill polymer membrane module for underwater applications, such as ocean gliders and autonomous sensors. The article describes the design of a prototype, and discusses the experimental results by comparison with a digital twin (analytical model and CFD model).

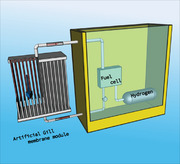

## 1 Introduction

Observational oceanography has recently seen an enormous increase in the use of autonomous underwater vehicles (AUVs) due to the ongoing technological advances in underwater robotics. In particular, for about two decades, a global network of profiling Argo floats^[^ ^1^ ^]^ and ocean gliders^[^ ^2^, ^3^ ^]^ has gathered a significant amount of in situ data of a large spectrum of processes that play out in the world's oceans and coastal seas, see, e.g., refs. [4, 5]. Both Argo floats and ocean gliders are AUVs propelled by a buoyancy engine, i.e., a volume‐changing device enabling the AUV to profile by either attaining positive or negative buoyancy. Floats make horizontal headway by drifting with the currents when “parked” at a certain depth, usually 1000 m. In the case of ocean gliders, wings convert a part of the vertical motion into a horizontal velocity component, see, e.g., refs. [6, 7]. These low‐power AUVs have an endurance of typically up to a few months (gliders), or a couple of years (floats).

Since Argo floats and gliders, and in fact all AUVs, are untethered, they require on‐board storage of energy. This is almost always in the form of batteries due to their technical simplicity, robustness and reliability. Which battery technology is preferred, depends on the specifics of the application, where, in practice, the choice is between high energy density (lithium primary cells) versus the low‐cost (alkaline cells) or the ability to recharge (lithium‐ion), see, e.g., refs. [8, 9] for comprehensive reviews on battery technology used in untethered underwater vehicles.

An attractive alternative technology for power conversion is provided by fuel cells, and in particular, Proton Exchange Membrane (PEM) fuel cells.^[^ ^10^ ^]^ PEM fuel cells use hydrogen and oxygen as reactants, directly generating electrical energy and water vapor. A review on the use of PEM fuel cells in unmanned aerial vehicles shows that a PEM fuel cell can achieve an energy density of up to three times that of lithium primary batteries using compressed hydrogen in advanced high‐pressure vessels and an energy density similar to lithium primary batteries when conventional metal hydrides – functional at room temperature – are used to store hydrogen.^[^ ^11^ ^]^ **Table** **1** provides approximate values for energy densities for various battery chemistries, as well as a fuel cell using metal hydrides for hydrogen storage.

**Table 1:** Typical energy densities.

Type	Energy density [W hr kg^−1^]	Rechargeable	Source
Lithium primary battery	≈350	No	^[^ ^8^ ^]^
Lithium‐ion battery	100 − 240	Yes	^[^ ^8^, ^9^ ^]^
Alkaline battery	≈140	No	^[^ ^8^ ^]^
PEM fuel cell (metal hydrides)	≈350	Yes	This study

Unfortunately, the application of unmanned aerial vehicles does not translate one‐to‐one to AUVs, where hydrogen storage, and in particular, oxygen storage pose technical hurdles.^[^ ^12^ ^]^ To date, several prototype AUVs have been developed that are powered by PEM fuel cells.^[^ ^13^, ^14^ ^]^ All these designs target fairly large AUVs and have in common that oxygen is stored on‐board, either in pressure vessels, or as chemical oxygen generators.

Pressure vessels that can store oxygen up to pressure levels of 30 or even 70 MPa require sufficiently thick walls to withstand the pressure. In particular, for smaller types of AUVs such as gliders, the weight and volume penalty becomes significant, lowering the system's energy density to non‐competitive levels compared with a battery solution based on lithium‐ion chemistry.

Chemical oxygen generators are another way to store oxygen on‐board. These oxygen generators use a chemical compound, usually chlorates or perchlorates of lithium, sodium or potassium, that evolve oxygen when heated.^[^ ^15^ ^]^ The use of chemical oxygen generators for small AUVs is not attractive for a number of reasons. To activate the chemical process the chemical compounds need to reach a high temperature of about 200 − 400 °C.^[^ ^15^ ^]^ For typical applications of chemical oxygen generators, such as emergency sources of oxygen used in aviation and mining, the high temperatures can be absorbed in the device itself, as the operational time is usually short. A slow release rate of oxygen seems problematic, as high temperatures are required for the duration of the use of the AUV (weeks). A significant weight penalty, and the fact that the generator cannot be recharged, but must be replaced involving potentially unsafe handling of highly oxidizing material,^[^ ^15^ ^]^ renders the chemical oxygen generator an undesirable solution for oxygen generation on small AUVs.

Instead of carrying oxygen on‐board, it can be harvested from the ambient water using an artificial gill system. The role of the artificial gill can be played by a membrane, in particular a polymer membrane. A polymer membrane is a flexible, but robust physical barrier that can separate different substances that are either in a liquid or gas phase, based on a solution/diffusion transport mechanism. There are numerous applications that have been reported in the literature indicating membranes to be capable of selective separation.^[^ ^16^, ^17^, ^18^ ^]^ In the case of artificial gill membranes, dissolved oxygen diffuses from water through the membrane into a gas phase supplying the fuel cell with oxygen. Using membranes to extract oxygen from the ambient water has been actively studied for several decades, but mainly focused on underwater life support systems. For example, results are reported on experiments that supported life of small animals (beetle, hamster, dog) underwater using artificial gills for a limited period of time.^[^ ^19^, ^20^ ^]^ The membrane modules in these studies were made with hollow fibers and flat sheet membranes from various materials. A further study shows how oxygen transfer can be improved by optimizing the arrangement of hollow fibers.^[^ ^21^ ^]^ Whether the water boundary layer limits the oxygen transfer, or the membrane itself, depends on the geometrical configuration of the membranes. In the engineered membrane modules the boundary layer is the predominant limiting factor,^[^ ^19^, ^21^ ^]^ whereas in fish gills, where the membrane surfaces are physically packed much closer together, the membrane's permeability ultimately determines the oxygen flux.^[^ ^22^ ^]^ A higher packing density, however, results in a higher energy requirement to force the flow past the membranes.

In this study, we propose a power supply system consisting of a PEM fuel cell with hydrogen stored in a metal hydride container combined with an artificial gill system that provides the oxygen required by the fuel cell and thereby eliminating the need to store oxygen on board. The weight and volume thus saved, can be used to store additional hydrogen. This system is rechargeable, which implies low running costs, and – most importantly – has the potential to achieve a similar or even higher energy density than currently possible with primary lithium batteries.

The aims of this work are, first to demonstrate the proof of concept by building a prototype system where the design parameters are to be derived from a mathematical model of the process of oxygen transfer, and second, to develop a digital twin by combining this mathematical model and a Computational Fluid Dynamics (CFD) model.

The proposed power supply system would primarily benefit the use of long‐endurance AUVs, such as ocean gliders, but it would also benefit other applications, such as autonomous underwater sensor systems. Examples are bottom‐mounted or moored underwater water quality monitoring sensors (underwater mining), where a limited energy content determines the effort and frequency of servicing and, ultimately, the operational costs.

## 2 Concept and Experimental Design

**Figure 1:**
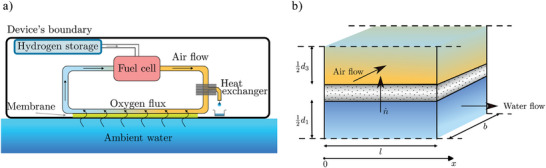
Panel (a) shows the conceptual picture of the power supply system. The fuel cell, as well as the circulating air‐flow, are contained within the device's boundary, such as a pressure vessel. The air‐flow interfaces the water flow with the mediation of a membrane. Oxygen within the circulating air‐flow is consumed by the fuel cell but replenished by the oxygen flux diffusing through the membrane. Hydrogen is supplied to the fuel cell from a low‐pressure metal hydride container. A passive heat exchanger dries the air‐flow sufficiently to prevent condensation in membrane section of the air channel. Panel (b) shows the definition of the idealized setting of an air‐flow and a water flow, driving an oxygen flux through the membrane. The dashed lines indicate planes of symmetry. The schematic is not drawn to scale.

The working concept of the system is shown in **Figure** **1**a. A PEM fuel cell generates electrical energy from the reactants hydrogen and oxygen. The hydrogen is stored in a container at low‐pressure using a metal hydride alloy that can store and release hydrogen at room temperature, hence combining safety with a sufficiently competitive energy density. The oxygen is provided to the fuel cell using a circulating air‐flow. A membrane surface, which is topologically part of the device's outer boundary (e.g., hull of an AUV), is permeable to oxygen that is dissolved in water, but inhibits the influx of liquid water (although water vapor still can permeate). The functioning of a membrane is based on the solution‐diffusion mechanism, meaning that gas molecules will diffuse and solubilize in the membrane in order to permeate through. This process is controlled by the relevant transport coefficients for the different gases, resulting in different permeabilities for the different gases.

The membrane interfaces the circulating air‐flow at its internal face, and the ambient water at its external face. The fuel cell's consumption of oxygen reduces the partial oxygen pressure in the circulating air‐flow, and when this partial pressure becomes lower than the partial pressure of the dissolved oxygen in the ambient water, an oxygen pressure gradient across the membrane develops. This pressure gradient drives the diffusive flux of oxygen through the membrane, and replenishes the internal air‐flow with oxygen, balancing the diffusive oxygen flux and the fuel cell's oxygen consumption rate.

Besides electrical energy, the fuel cell also produces waste heat and water vapor. When the warm moist air leaving the fuel cell gets into contact with the colder membrane surface, some of the vapor may be expected to condense and potentially hamper the air circulation. Therefore an additional component is mandatory that sufficiently dries the air before it passes the membrane. A passive heat exchanger, exploiting the temperature difference between the internal circulating air and the external ambient water would be sufficient. Although essential for an optimally functioning system, such a heat exchanger was not integrated into the prototype, because it is not to be expected that the limited amount of water vapor produced due to relatively short experimental running times of a few hours, will render the membrane module ineffective.

Whereas the fuel cell and hydrogen storage using metal hydrides are system components that can be sourced commercially, the key component of the system is a membrane module mimicking fish gills capable of extracting oxygen from the ambient water. It is mainly the efficiency of the membrane module that determines whether or not a sufficiently large oxygen flux can be achieved to allow the fuel cell to generate the required power output for an ocean glider (AUV or any other underwater measurement system) to function. This does not mean that the other components do not require technical improvement. Any improvement of such components would lead to an increase in endurance but would have no significant effect on the amount of power a fuel cell could generate.

### 2.1 A Mathematical Model of Oxygen Transfer Across a Membrane

To facilitate the explanation of the working concept, Figure 1a depicts the membrane surface as a single surface. Instead of a single membrane surface, we consider in this work a stack of membrane surfaces, which creates a system of interleaving water and air channels separated by membranes. We start with analyzing such a system of stacked membranes in its simplest form: an air‐flow and a water flow separated by a membrane, as shown in Figure 1b. A relationship is established between $p_{1}\left(0\right)$, the partial oxygen pressure entering the water channel at x=0, $p_{3}\left(l\right)$, the partial oxygen pressure leaving the air channel at x=l, and $\dot{n}$, the molar oxygen flux across the membrane for steady‐state conditions. This relationship, derived in Appendix A, reads

(1) $$p_{3}\left(l\right)\approx p_{1}\left(0\right)-\dot{n}\left(c_{1}+c_{2}+c_{3}\right)$$

with

(2) $$\begin{array}{cc} c_{1} & ={\left(2H_{\text{cp}}\;b\;l\;h_{1}\right)}^{-1} \end{array}$$

(3) $$\begin{array}{cc} c_{2} & ={\left(P\;l\;b\right)}^{-1} \end{array}$$

(4) $$\begin{array}{cc} c_{3} & =\frac{R\;\Theta }{Q_{3}} \end{array}$$

Herein *h*_1_ is the transfer rate of oxygen in the water channel (perpendicular to the flow), *P* the membrane permeance (equal to the membrane permeability divided by the membrane thickness), *l* the length and *b* with width of the membrane/channel, *Q*_3_ the flow rate in the air channel, *R* the gas constant and Θ the absolute temperature. The parameter *H*_*cp*_ is Henry's coefficient of solubility for oxygen in water, and relates the molar concentration of oxygen to the partial pressure of the dissolved oxygen. The transfer rate *h*_1_ can be related to the coefficient of diffusivity of oxygen in water, 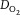 through the Sherwood number^[^ ^23^ ^]^

(5)

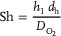

where *d*_h_ is the hydraulic diameter. For a high‐aspect‐ratio rectangular channel (*b* ≪ *d*_1_), we have *d*_h_ = 2*d*_1_, where *d*_1_ is the height of the water channel. Equation (1) shows that the oxygen flux through the membrane is proportional to the partial oxygen pressure difference across the membrane, $p_{3}\left(l\right)-p_{1}\left(0\right)$. The proportionality factor can be interpreted as a mass transfer resistance, due to the lateral transport of oxygen in the water channel, the oxygen flow through the membrane and the lateral transport of oxygen in the air channel, analogous to a configuration of three electrical resistors connected in parallel.

To put this model in context, we consider a typical ocean glider that has a power consumption of about *P*_g_ = 5 W on average, with some variability depending on the sensor suite and sampling strategy. The required molar rate of oxygen is given by $\dot{n}=\frac{1}{2}P_{g}/w$, where *w* is the amount of work per mole hydrogen that is effectively available for electrical energy and the factor 1/2 appears because for each mole of hydrogen only half a mole of oxygen is required. We can relate *w* to the reaction's enthalpy, Δ*H*, and the fuel cell's efficiency η as η = *w*/Δ*H*. The fuel cell enthalpy equals Δ*H* = 284 kJ mol^−1^, see, e.g., ref. [24]. Assuming a realistic fuel cell efficiency of η = 0.55, it is found that the fuel cell would require a molar rate of oxygen of $\dot{n}\approx 16\;\mu \text{mol}\;s^{-1}$. Allowing the partial oxygen pressure in the air channel to drop to about 0.1 bar, the required surface area of the membrane can be estimated by substituting reasonable values for the remaining parameters: *h*_1_ = 2.4 × 10^−6^ m s^−1^ (corresponding to Sh = 8, Table 8.1 in ref. [23]), *H*_*cp*_ = 1.3 × 10^−5^ mol m^−3^ Pa^−1^, 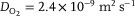, *R* = 8.31 Pa m^3^ mol^−1^ K^−1^, Θ = 293 K, *l* = *b* = 0.20 m, *d*_1_ = 4 × 10^−3^ m, and *d*_3_ = 3 × 10^−3^ m. Furthermore, the membrane's permeance is set to *P* = 3.39 × 10^−9^ mol m^−2^ s^−1^ Pa^−1^ (See Membranes in Experimental Section). For now, *Q*_3_ is assumed to be sufficiently large, so that *c*_3_ ≪ *c*_1_. With this parameter setting, the required surface area is approximately 3 m^2^. In addition, the ratio *c*_1_ to *c*_2_ evaluates to about 54, meaning that the oxygen permeate flux is predominantly controlled by the convective oxygen flux in the water flow.

### 2.2 Prototype Design

**Figure 2:**
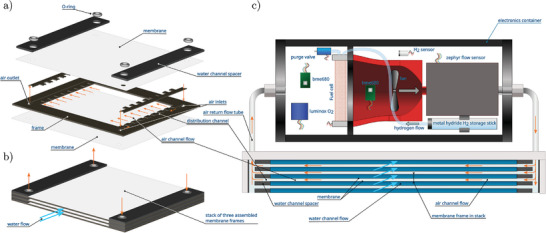
Schematic representation of the experimental setup. Panel (a) shows the individual components that make up a single membrane frame assembly. Panel (b) shows a stack of three such assembled membrane frames by means of example. The schematic in panel (c) shows the top five frames fitted in the membrane module, stacked as shown in the bottom left‐hand side panel. The cylindrical electronics container is mounted on top of the membrane module and is connected to the air channels inside the membrane module by stainless steel tubes. The electronics container houses the fuel cell, hydrogen storage, sensors and an air fan, the latter drives the internal air circulation. The total number of assembled membrane frames used is 38.

The prototype membrane module is designed as a stack of multiple membrane frame assemblies. A drawing of the parts of a frame assembly is shown in **Figure** **2**a. The basis is a 3D‐printed polylactide frame. Flat‐sheet membranes based on poly(octylmethylsiloxane),^[^ ^25^, ^26^ ^]^ see also Membranes in Experimental Section, are glued to either side of the frame, thus creating an air channel with height *d*_3_ = 3 mm. The membranes are supported by a polypropylene grid spacer (not shown), preventing the membranes from collapsing. Each frame has two pairs of holes, labeled inlet and outlet. When stacked, the holes align and create vertical inlet and outlet channels spanning the stack. O‐rings are used to ensure sealed connections. For each frame, both the inlet and outlet openings, respectively, are connected by a channel – each with 8 openings facing inward – that distributes part of the vertical air‐flow horizontally, thus creating a horizontally uniform air‐flow between the membrane sheets, as shown by the orange arrows. Two spacer strips are placed on top of the upper membrane so that, when another frame assembly is placed on top, this creates a water channel with height *d*_1_ = 4 mm. The schematic shown in Figure 2b depicts a stack of three of such frame assemblies. The active area of each membrane is equal to *l* × *b* = 0.2 × 0.2 m^2^. A total of 38 membrane frames were stacked to form the module with a total membrane surface area of 3.0 m^2^.

The complete system is schematically shown in Figure 2c, see also Figure S1 (Supporting Information). The membrane stack, of which only the top five frames are shown, is fitted in a cube‐shaped container with a linear size of approximately 30 cm. Centered in the front and back face of the cube are a circular water inlet and outlet, both with a diameter of 6 cm. An external pump with an adjustable flow rate is used to achieve a constant flow rate through the membrane module.

A separate electronics container houses the fuel cell, a metal hydride cartridge storing up to 1 g of hydrogen gas, a fan to drive the air circulation, and a range of sensors to measure partial oxygen pressure, total pressure, temperature, humidity and air‐flow rate, see Figure 2c. Details on the sensors used is given in the Experimental Setup section of the Experimental Section.

The air inlet/outlet of the electronics container connects to the air outlet/inlet of the membrane module container, thus establishing the circulating air channel (cf. Figure 1a). The fuel cell is connected to a configurable load of resistors with which the output current can be set. A digital current sensor, included in the sensor circuitry, measures the electrical current generated by the fuel cell. The sensor circuitry is powered independently from the fuel cell and outputs a data stream logged by a computer.

### 2.3 Experimental Design

To formulate the experimental program, it is convenient to cast the mathematical model given in Equation (1) into a non‐dimensional form. Since *c*_1_/*c*_3_ ≈ 28, see Appendix A, the reduction in the oxygen flux due to the lateral transport of oxygen in the air channel can be neglected. Even though *c*_1_/*c*_2_ ≈ 56, we choose to retain the effect of the membrane itself, however, anticipating the analysis of the experimental results below. This analysis suggests that the value of *P* of the membranes used in the experimental setup could be significantly smaller than initially assumed, rendering *c*_1_/*c*_2_ ≈ 7.

Consistent with the scaling analysis invoked below, the following four parameters are used to scale all the terms in Equation (1):

(6) $$\begin{array}{ccc} & & \left[\Delta p\right]=\text{kg}\;m^{-1}\;s^{-2},\;\left[H_{\text{cp}}\right]=\text{mol}\;{\text{kg}}^{-1}\;m^{-2}\;s^{2},\\ & & \left[\nu \right]=m^{2}\;s^{-1},\;\left[d_{1}\right]=m \end{array}$$

where ν is the kinematic viscosity. Setting Δ*p* = $p_{1}\left(0\right)-p_{3}\left(l\right)$), using Equation (5) to eliminate *h*_1_ and introducing the Schmidt number 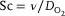, Equation (1) can be written in non‐dimensional form as

(7) $${\dot{n}}_{*}=\frac{b\;l}{d_{1}^{2}}\;{\left(\text{Sc}\;{\text{Sh}}^{-1}+P_{*}^{-1}\right)}^{-1}$$

where ${\dot{n}}_{*}$ and P_*_ are the non‐dimensional oxygen permeate flux and the non‐dimensional membrane permeance, respectively, given by

(8) $$\begin{array}{cc} {\dot{n}}_{*} & =\frac{\dot{n}}{\Delta pH_{\text{cp}}\nu d_{1}} \end{array}$$

(9) $$\begin{array}{cc} P_{*} & =\frac{Pd_{1}}{H_{\text{cp}}\nu } \end{array}$$

Equation (7) may seem to suggest that the oxygen permeate rate is independent of the water flow rate. However, the application of a scaling analysis (Pi‐theorem)^[^ ^27^ ^]^ can be used to demonstrate that the Sherwood number must be a function of (at least) the Reynolds number characteristic for the water flow (Appendix B). Since the design of the experimental setup does not allow for physical changes (constant *d*_1_, *b*, *l* and *P*), and also, the properties of the water are fixed (constant *H*_cp_, Sc and ν), the only parameter that can be varied is the Reynolds number, and thus the Sherwood number, by varying the flow rate through the membrane module.

Besides setting the flow rate, we also need to apply an electrical load to the fuel cell for the latter to work. Depending on the load, the fuel cell generates an electrical current *I*. The fuel cell's oxygen consumption rate ${\dot{n}}_{\text{fc}}$ can be related to *I* using the empirical relationship given by Equation (13), see also the Membrane Module Oxygen Permeate Flux section in the Experimental Section. This implies that the equilibrium value for $\dot{n}$ is dictated by the electrical load applied. However, for a constant flow rate and at equilibrium conditions, Equations (7) and (8) indicate that any change in $\dot{n}$ is compensated by a change in Δ*p*. Hence, three experiment configurations are defined, labeled I, II and III, where each label represents a flow rate. For each configuration, three replicate experiments are defined, of which two are duplicates, i.e., having the same setting for *I*, and one having a setting for *I* that is either halved or doubled. The experimental configuration matrix is summarized in **Table** **2**.

**Table 2:** Measurement matrix: three experiments {I, II, III}, each with a different water flow rate generated by an external pump (*Q*_pump_), times three replicates {1, 2, 3}, each with a preset electrical current generated by the fuel cell.

	Exp. I	Exp. II	Exp. III
	*Q*_pump_ = 0.478 ℓ s^−1^	*Q*_pump_ = 0.663 ℓ s^−1^	*Q*_pump_ = 0.847 ℓ s^−1^
**1**	*I* = 15.6 mA	*I* = 15.8 mA	*I* = 16.3 mA
**2**	*I* = 15.7 mA	*I* = 42.3 mA	*I* = 16.9 mA
**3**	*I* = 28.7 mA	*I* = 46.6 mA	*I* = 29.2 mA

## 3 Results

**Figure 3:**
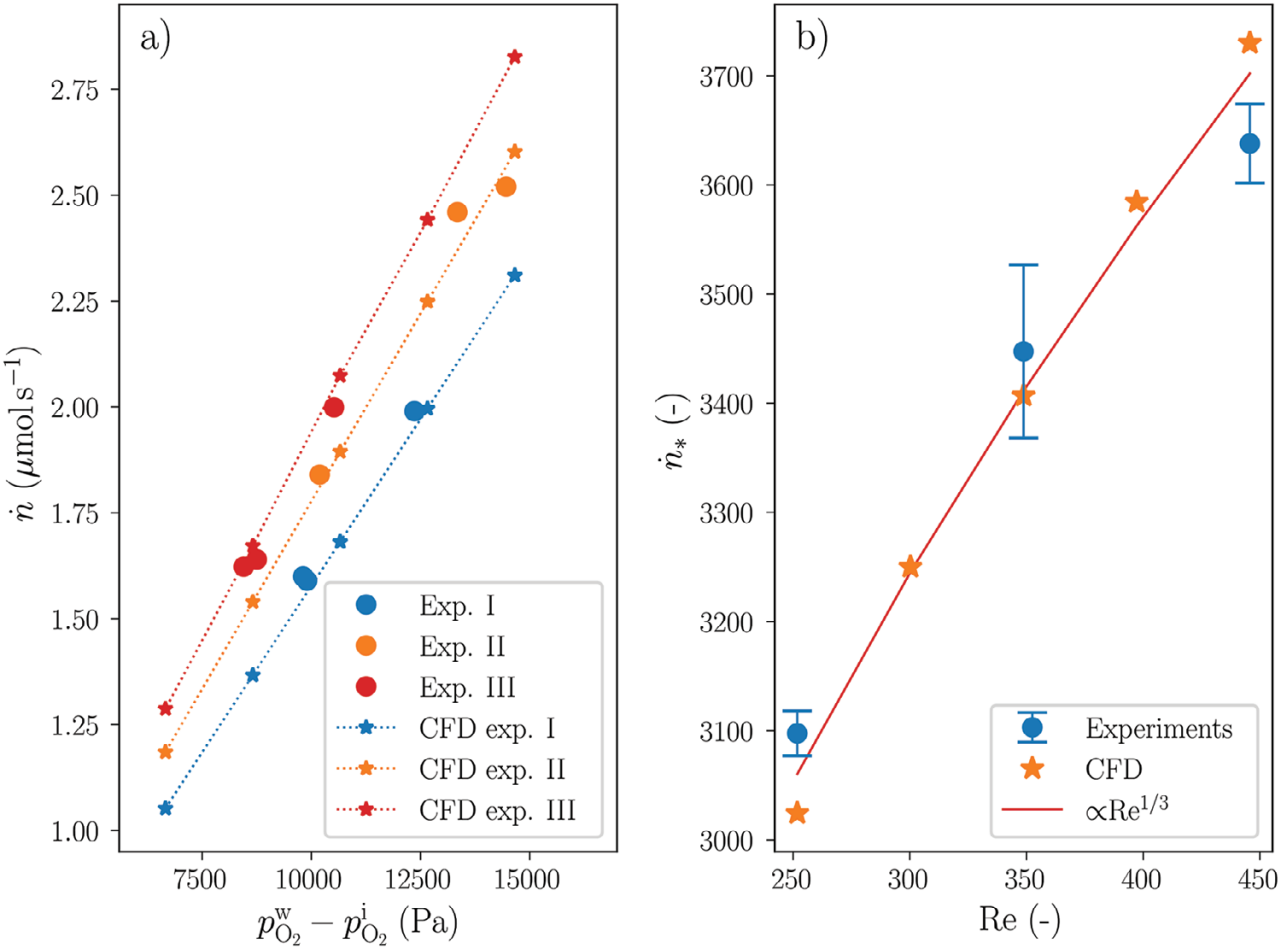
Panel (a) shows the measurement and CFD data, expressed as the oxygen permeate flux versus the difference of the partial pressure of dissolved oxygen in water, measured at 17660 Pa, and the partial oxygen pressure measured inside the system. The solid dots refer to the measurements, whereas the star symbols represent the results from the corresponding CFD simulations. An overview of the experimental settings is given in Table 2. Panel (b) shows the same data expressed in non‐dimensional variables. All experiments are now represented by the average of their replicates (blue solid dots, sample size *n* = 3). The error bars correspond to the standard deviation, computed from the three replicates. The orange star symbols represent the results of the CFD model. The red curve is proportional to Re^1/3^, scaled by fitting to the experimental data.

The experimental results are shown in **Figure** **3**a, where each data point corresponds to a measurement taken when a steady‐state condition of a particular experiment listed in Table 2 had been reached. The solid circles show the measured oxygen permeate flux as a function of the difference of the partial pressure of dissolved oxygen in water and the internal partial oxygen pressure (as measured inside the electronics container). The partial pressure of dissolved oxygen, 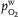, was determined at 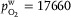 Pa using a hand‐held dissolved oxygen measurement device (see the Membranes section in the Experimental Section). The colors blue, orange and red correspond to the experiments I, II, and III, respectively.

The same measurement data are shown in non‐dimensional space in Figure 3b as an average of the three replicates of each experiment (blue solid circles). Here, *H*_cp_, and *d*_1_ are set as above and ν = 1 · 10^−6^ m^2^ s^−1^. The pressure drop across the membrane Δ*p* is computed from 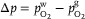. Herein, 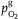 is the (average) partial oxygen pressure inside the membrane module. For practical reasons it is assumed that 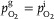. Furthermore, the Reynolds number is computed from the flow rate of the pump according to

(10) $$\text{Re}=\frac{Q_{\text{pump}}\;d_{1}}{A_{\text{eff}}\;\nu }$$

where *A*_eff_ is the effective inflow area. This area is computed from *A*_eff_ = *rN* *d* *b*, where *N* is the number of water channels (*N* = 38) and *b* the channel width (*b* = 0.2 m). The parameter *r* is a reduction factor, set to *r* = 0.25 to account for the inhomogeneous flow conditions inside the membrane module, as will be discussed shortly.

A CFD model, simulating the flow through the membrane module using a solver for transient flow using the low Reynolds turbulence model *k*‐*k*_l_‐ω^[^ ^28^, ^29^ ^]^ and a tracer solver for the oxygen transport was developed to facilitate the interpretation of the measurements. The tracer solver was modified to implement an appropriate boundary condition applied to the membrane boundaries. Further details are given in the CFD Model section of the Experimental Section.

**Figure 4:**
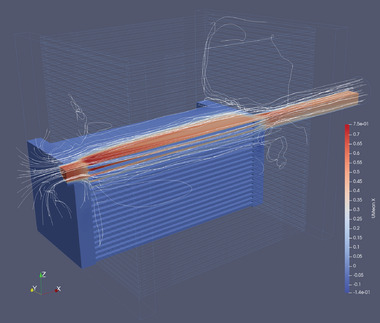
Snapshot of the flow field of the velocity component in *x* direction for experiment II (*Q*_pump_ = 0.663ℓ s^−1^), illustrating the inhomogeneity of the flow field. The velocity field is shown for one quadrant of the membrane module only, whereas the (wet) outline of the whole membrane module is visible. The streamlines are seeded on a vertical line through the center of the circular water inlet of the membrane module.

The flow fields corresponding to experiments I, II and III as computed by the CFD model, reveal that the larger part of the flow is concentrated around the center axis of the membrane module. **Figure** **4** shows a snapshot of the flow field and corresponding streamlines. We observe that most streamlines follow a path through a relatively narrow core in the center of the module. It is estimated that the volume that is actively flushed to be roughly 25% of the total membrane module's volume, hence *r* = 0.25.

Flow fields, averaged over 60 s, are used as forcing for the tracer simulations that are solved for a range of values for the internal partial oxygen pressure. The oxygen permeate flux, computed for equilibrium conditions, is shown in Figure 3a by the curves with star symbols and in Figure 3b by the orange star symbols.

## 4 Discussion

Past work with membrane modules has shown that they can function as artificial gills and sustain small animals underwater.^[^ ^19^, ^20^ ^]^ In this work we demonstrate that a membrane module can also be used to provide oxygen to a fuel cell operating underwater: the experiments carried out show that an equilibrium can be reached where the oxygen consumption by a fuel cell is balanced by the membrane module's uptake of oxygen from the ambient water.

The measurements, presented in dimensional space (Figure 3a), show that a higher difference in partial oxygen pressure across the membrane results in a higher oxygen permeate flux. Furthermore, a higher water flow rate through the membrane also results in a higher oxygen permeate flux. The CFD model, set up in an attempt to account for all the complexities present in the physical experiment, produced results that compare well with the measurements and show the same features. This is an important achievement, as it demonstrates the quantitative predictive capabilities of the CFD simulations.

It is noted, however, that the membrane permeance was used as a tuning parameter. With a value of *P* = 3.39 × 10^−9^ mol m^−2^ s^−1^ Pa^−1^, determined experimentally, see Membranes in Experimental Section, the CFD model overestimates the oxygen permeate flux by a factor of ≈1.5. The results shown were computed with a reduced value of *P* = 2.6 × 10^−10^ mol s^−1^ m^−2^ Pa^−1^ (see the CFD Model section of the Experimental Section). The significant reduction in the permeance by a factor of 12.9 may be caused by condensation droplets on the membranes' surfaces that hamper the oxygen transfer through the membranes and therefore lowering the effective membrane permeance. Although we have not a direct observation of this, as it would mean that the membrane frames had to be destroyed, water droplets were found inside the stainless steel tubing. A passive heat exchanger, referred to in Section 2, would mitigate this.

To discuss the features of the measurement data and CFD model results more specifically, we can consider these in the light of the mathematical model developed in Section 2.1. Here, it was argued that the current experimental setup allows the variation one model parameter only, namely the Reynolds number. Hence, the mathematical model should be able to make also one testable prediction. In dimensional space, Equation (1) suggests that for a given flow condition (*h*_1_ is constant), the oxygen permeate flux is proportional to Δ*p*, where Δ*p* = $p_{1}\left(0\right)-p_{3}\left(l\right)$) as before.

The CFD results, presented in dimensional space, clearly show a linear relationship between $\dot{n}$ and Δ*p* for each experiment, where we take 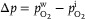. Additionally, it is found by extrapolation that the linear curves converge for all experiments in the origin, hence, $\dot{n}\propto \Delta p$. Due to the limited amount of data points and the experimental error, extrapolation of the measurement data is not considered meaningful. However, the argument can be made that the measurement data are well represented by the CFD model results and we can infer from this that the measurements also adhere to the proportionality $\dot{n}\propto \Delta p$.

Equation (7) suggests that ${\dot{n}}_{*}$ is a function of Sh. We also argued that Sh = *f*(Re, …), so that with all other parameters being constant for the current experimental setup, ${\dot{n}}_{*}$ must be a function of Re only. Indeed, the data points of all CFD simulations for given Re collapse into a single data point in non‐dimensional space (Figure 3b). Interpreting the magnitude of the error bars to reflect the experimental error, then it appears that the experimental data also seem to collapse onto one point for each experiment. Hence, the experimental data support the prediction of the simple analytical model that ${\dot{n}}_{*}$ is a function of Re. Indeed, using Python's Scipy module 1.11.1, an ANOVA test revealed a significant difference among the results of all three experiments {I, II, III} (*p* = 1.3 × 10^−4^), and a post‐hoc Tukey HSD test was conducted to determine pairwise differences. The results of the Tukey test are summarized in **Table** **3**.

**Table 3:** Results of the post‐hoc Tukey HSD test comparing the mean values of cases I, II, and III. Significant differences between case pairs are indicated by *p*‐values below 0.05.

Comparison	*p*‐value
I vs II	1.2 × 10^−3^
I vs III	1.1 × 10^−4^
II vs III	2.4 × 10^−3^

In order to investigate the dependency of ${\dot{n}}_{*}$ on Re, we turn to how Sh depends on Re. For fully developed flow conditions in a high‐aspect‐ratio rectangular channel, Sh is expected to attain a constant value of about 8.^[^ ^23^ ^]^ It can be shown that the flow can be considered hydrodynamically developed, that is, entry effects are negligible. Because of the non‐zero oxygen flux at the channel boundaries, the oxygen concentration profile will generally not be constant downstream the water flow. However, the shape of the profile might be expected to become self‐similar after some distance from the channel point of entry. Analogously to the process of heat transfer, the flow can be considered “thermally” developed if (Re Sc *d*_1_/*l*)^1/3^ < 2.^[^ ^23^ ^]^ In the present context, the criterion would evaluate to about 12, meaning that the oxygen concentration profile is not fully developed. For not fully “thermally” developed flow conditions an empirical function similar to

(11) $$\text{Sh}=1.86{\left(\frac{\text{Re}\;\text{Sc}}{l/d_{1}}\right)}^{1/3}$$

has been suggested (e.g. Table 8.4 in ref. [23]).

From substituting Equation (11) into Equation (8) it follows that the mathematical model predicts that ${\dot{n}}_{*}\propto {\text{Re}}^{1/3}$ for *c*_1_/*c*_2_ ≪ 1. This proportionality, scaled to fit the measurements, is shown by the red curve in Figure 3b. Indeed, the relationship between $\dot{n}$ and Re is similar for both the measurements and the CFD model, and is echoed by the mathematical model's prediction. From this we can draw two further important conclusions. The CFD model setup is capable of reproducing the measurements for different flow conditions, and both the measurements and the CFD model results are shown to be in line with the qualitative predictions made by the mathematical model. With these two tools we can not only understand the processes involved but also make quantitative predictions of new membrane module designs.

In Section 2.1 an ocean glider was used as a fictive host for the proposed power supply system. The required permeate flux was expected to be $\dot{n}=16$ µmol s^−1^, corresponding to a power output of approximately 5 W. Such a permeate flux could be achieved by a membrane module with 3 m^2^ surface area. Depending on how low the internal partial oxygen pressure was allowed to fall, the power delivered by the fuel cell was in the range 0.18 – 0.3 W, where the membrane module achieved an oxygen permeate flux of 1.5–2.5 µmol s^−1^, see also Figures S2–S4 (Supporting Information). The achieved permeate flux was roughly a factor 6 – 10 lower than the target permeate flux. Is this a serious problem? We argue it is not.

The mathematical model is based on the assumption of homogeneous flow through all the water channels. However, the consequence of the module being designed as a desktop demonstrator, with a small water inlet and outlet, is that the internal flow pattern was less homogeneous than assumed, as is shown by the flow fields calculated with OpenFOAM's transient flow solver (Figure 4). The deviation from the flow patterns of the prototype and the idealized setup, depicted in Figure 1b, has a detrimental effect on the magnitude of the oxygen permeate flux. However, it also poses a challenge for the CFD model that is considerably larger. The fact that the CFD model performed so well is very encouraging and provides confidence in the application of CFD modeling to other complex geometries in future work.

The mathematical model provides a way to estimate which design parameters could be changed to increase the oxygen permeate flux. For example, reducing the length of the membrane by a factor of *f* – but keeping the total membrane area constant – suggests an increase of the permeate flux by a factor of *f* ^1/3^. Similarly, reducing the channel heights would improve $\dot{n}$. In general, a higher oxygen permeate flux per unit membrane surface can be obtained if the membranes were made smaller and packed closer together than was done in the prototype. In fact, the membrane module would then look much more like the gills of fish, which consist of a large number of small lamellae, packed closely together.^[^ ^30^ ^]^ The lamellar dimensions, and in particular the interlammelar distance (cf. *d*_1_) in fish are shown to be optimal as a result of evolutionary pressure.^[^ ^31^ ^]^ To construct a membrane module from membrane frames with dimensions of the order of fish lamellae may be beyond what is possible with current manufacturing techniques for polymer membranes, however.

The integration of the proposed power supply system in a real‐world application such as an ocean glider raises a number of new, mostly engineering, challenges. The space freed‐up by removing batteries will be taken up partially by metal hydride containers to store the hydrogen. Because the internal pressure inside a metal hydride container is relatively low (≈1 MPa), shaping such containers is subject to less stringent constraints than high‐pressure vessels, allowing to make efficient use of the available internal space. From a thermodynamics point‐of‐view, it makes sense to integrate the fuel cell into the metal hydride container design, where heat of the fuel cell can be used to raise the temperature of the metal hydride. Besides electricity, fuel cells generate water vapor and therefore increasing the humidity. Levels of humidity that are too high may lead to condensation of water vapor inside the membrane module and reducing the oxygen permeate rate. A heat exchanger, for example, could be implemented to reduce the humidity such that condensation does not occur. For buoyancy‐driven AUVs, such as gliders, an additional mechanism is required to periodically expel a small amount of condensed water, so that the vehicle does not gain too much weight.

The biggest engineering challenge may be the integration of the membrane module. It is important that the design does not adversely effect the hydrodynamics significantly. Since access to ambient sea water is required, a placement in a flooded hull section is preferred. Some amount of energy is going to be required to drive the flow through the membrane module, either as a result of dynamic pressure gradient or by using a pump, and to run additional electronics to control the power supply system. Furthermore, some accumulator of electrical energy may be required (rechargeable battery, or super capacitor) to deliver high electrical currents for short periods of time to drive a buoyancy pump.

The system is expected to work well in most oceanic and coastal waters, which are well enough oxygenated, in particular the surface mixed layer (upper 200 m or so). In some regions, such as the eastern ocean boundary layers (tropical east Pacific and topical east Atlantic), however, hypoxic conditions might occur below the mixed layer,^[^ ^32^ ^]^ where the partial oxygen pressure in the water may be too low to drive a sufficient oxygen flux. For profiling systems such as gliders, the system still could work, provided that the system spends a sufficient amount of time in the surface mixed layer and the internal air volume is large enough to act as an oxygen buffer.

## 5 Conclusion

This study presents a proof‐of‐concept, demonstrating successfully that a fuel cell can be run underwater whilst obtaining the required oxygen using a polymer membrane. The mathematical model and the CFD model we have developed are the tools that are needed to design and build an improved membrane module: the mathematical model provides us with process knowledge and the CFD model provides us the ability to make quantitative predictions. Preliminary calculations show that it is technically possible to devise a power system suitable for low‐power underwater robots (ocean gliders) and instruments based using hydrogen fuel cell technology, and has an energy density equal to or possibly exceeding that of primary lithium batteries.

## 6 Experimental Section

### Experimental Setup

The experimental setup consisted of four key components: a fuel cell, the hydrogen storage device, the membrane module, and a compartment with sensors for monitoring the system. A 12 Watt fuel cell (H‐12 PEM fuel cell) was used. Hydrogen was stored in metal hydride containers (Hydrostik Pro). A solenoid purge valve was used to purge the fuel cell at regular intervals to remove any impurities that may build up inside the fuel cell. All three components were sourced from Horizon Fuel Cell Europe, s.r.o (Prague, Czech Republic).

The sensors in the electronics compartment were controlled and logged by an Arduino UNO (Mouser Electronics Inc, Munich, Germany). Temperature, pressure and humidity were measured using a BME680 break‐out board (Mouser Electronics Inc, Munich, Germany), oxygen pressure was measured with a Luminox LOX‐O2 sensor (Angst+Pfister and Power AG, Zürich, Switzerland). The air‐flow rate was measured with a Honeywell Zephyr Digital Airflow Sensor (15 SLPM) (Mouser Electronics Inc, Munich, Germany). The fan, used to circulate the internal air, was originally integrated in the fuel cell, but for this experimental setup it was removed and placed in its own housing. The dissolved oxygen concentration in the water basin was measured using a handheld measurement device) (Greisinger, GMH‐3611, Remscheid, Germany).

An Arduino Mega (Mouser Electronics Inc, Munich, Germany), placed outside of the system, interfaced the Arduino UNO via a 2 m waterproof cable and connectors (SubConn Micro Circular 6 pin connectors (Bornhoeft Industriegeräte GmbH, Kiel, Germany)), receiving the measured data over a serial line at 1 s intervals. An INA219 current sensor break‐out board (Mouser Electronics Inc, Munich, Germany) was used to measure the current and voltage output of the fuel cell. The Arduino Mega also controlled the air‐flow rate by setting the fan speed. A circuitry of high‐power resistors acted as the fuel cell's electrical load. All sensor data were assembled and outputted on serial line to be logged by a computer.

Both the internal and the external electronic circuits were powered using an external power supply, and the fuel cell and its load circuitry were electrically isolated.

### Membranes

The membranes were made of poly(octylmethylsiloxane) (POMS), a typical polysiloxane hydrophobic polymer used extensively in gas separation applications.^[^ ^25^, ^26^ ^]^ POMS membrane has a rubbery character, giving POMS suitable (selective) permeability for oxygen (in relation to other gases like nitrogen or water vapors) compared to other commonly used membrane materials for underwater applications like polytetrafluoroethylene (PTFE), or polyolefin‐type materials (e.g., polyethylene).^[^ ^33^ ^]^ Poly(dimethyl siloxane) (PDMS) is also a possible material that could be used. However, the lower amount of methyl groups on the sides of the polymer backbone in comparison to POMS decreases the hydrophobicity so that, under the same conditions, water vapors permeate at a significantly faster rate based on the solution‐diffusion mechanism of the membrane function. Furthermore, the permeability for oxygen for PTFE or polyolefin‐type membranes is significantly lower than that of POMS.^[^ ^34^ ^]^

The POMS membranes used in the experimental setup were made in‐house. To that end, a precursor solution was used for the membrane casting, consisting of siloxane‐based crosslinking and reinforcing agents, and a platinum‐based catalyst for the thermal crosslinking reaction, while isooctane is used as the solvent. The exact composition of the precursor solution cannot be disclosed due to commercial reasons (further information can be made available by the authors upon reasonable request). The casting was done on a porous non‐woven support that was coated with a thin layer of polyacrylonitrile with the use of the doctor blade technique.^[^ ^25^ ^]^ The final membrane has a thickness of ≈4 µm. The permeance of the membrane was determined at *P* = 3.39 × 10^−9^ mol m^−2^ s^−1^ Pa^−1^, using a custom‐made gas‐permeation device on four separate membrane sheet samples.

### Membrane Module Oxygen Permeate Flux

The oxygen permeate flux was computed using a budget calculation, where the rate of change of the amount of oxygen inside the system was balanced by an influx of oxygen through the membranes and outflux of oxygen due to the consumption of the fuel cell. Invoking the ideal gas law, it can be written

(12)

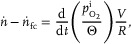

where *V* the total internal volume of air, composed of the internal air volume of the membrane system (including tubing) and the volume of the electronics container.

All experiments, except for experiment II/1 (Table 2), reached an equilibrium. This means that for these experiments, the right‐hand side of Equation (12) evaluates to zero, so that $\dot{n}$, for a given value of 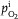, can be evaluated from $\dot{n}={\dot{n}}_{\text{fc}}$. Unfortunately, experiment II/1 ran out of hydrogen supply before an equilibrium was reached. For this experiment, the oxygen flux was quantified taking into account the right‐hand side term of Equation (12), by estimating the averaged rate of change in 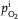 over a 1800 s period, where 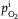 was still roughly 1 kPa below the expected equilibrium value.

The evaluation of the oxygen budget requires the internal air volume and the fuel cell's oxygen consumption rate to be quantified. The fuel cell's oxygen consumption rate was not measured directly, but can be calculated from the electrical current generated, based on the expectation that for ideal fuel cells ${\dot{n}}_{\text{fc}}$ is proportional to *I*.^[^ ^24^ ^]^ In practice, inefficiencies due to internal currents can occur. Furthermore, whilst a higher than atmospheric partial oxygen pressure is known to improve the fuel cell's efficiency,^[^ ^24^ ^]^ a lower than atmospheric partial oxygen pressure may have detrimental effects on the fuel cell's efficiency. To account for a constant fuel cell inefficiency, and the detrimental effects of a low partial oxygen pressure, an empirical relationship for ${\dot{n}}_{\text{fc}}$ is defined, given by

(13)

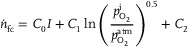

The middle term accounts for any effect of the internal partial oxygen pressure on the efficiency of the fuel cell and the last term accounts for a constant inefficiency.

**Figure 5:**
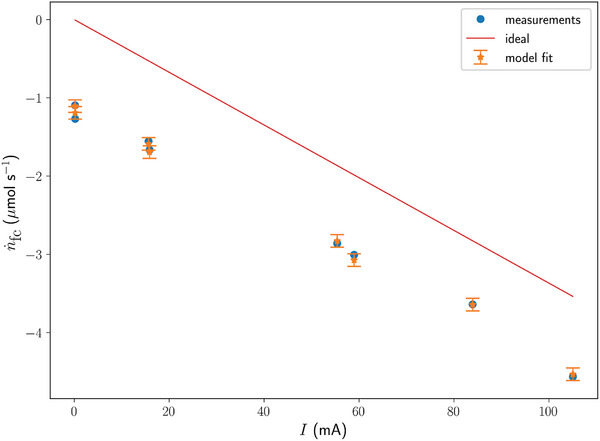
Measurements of the rate of oxygen consumption by the fuel cell as a function of the electrical current. The red curve shows the ideal behavior. The blue dots represent the measured values of the oxygen consumption rate. The orange stars represent the oxygen consumption rate, computed from the electrical current using the empirical relation (13), with a standard deviation of the residuals of σ = 0.03 µmol s^−1^, computed from a sample size of *n* = 8.

The coefficients *C*_*i*_ were determined from experiments with the membrane module disconnected. Then, in Equation (12), $\dot{n}=0$ and *V* is equal to the electronics container's internal volume *V*_c_, determined experimentally at *V*_c_ = 2.21 × 10^−3^ ± 0.01 × 10^−3^ m^3^, see below. Using Equation (12), ${\dot{n}}_{\text{fc}}$ can be determined for a range of preset values of *I* and for range of values of 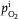. The coefficients *C*_*i*_ are quantified using a regression analysis (**Figure** **5**), resulting in *C*_0_ = 3.25 × 10^−5^ mol C s^−2^, *C*_1_ = 2.24 × 10^−7^ mol s^−1^, and *C*_2_ = 1.19 × 10^−6^ mol s^−1^, while the standard deviation of the residuals amounts to 0.03 µmol s^−1^.

The determination of the coefficients *C*_*i*_, as well as the evaluation of Equation (12) requires the internal air volume of the system without and with the membrane module, respectively. Since a significant fraction of the internal volume of the electronics container was occupied by electronic devices and mounting constructions, its internal volume cannot easily be calculated accurately. To quantify *V*_c_ experimentally, the membrane module was replaced with a container with gas‐impermeable walls and of known volume *V*_t_. At the start of the experiment, the total air pressure was equalized to atmospheric pressure, which was measured. Two valves were set such that both containers were disconnected. A moderate vacuum was then applied to the electronics container, creating a pressure difference between the containers. The increase in the total air pressure, after opening one of the values allows *V*_c_ to be computed from *V*_t_ by applying the ideal gas law.

The total volume of the system was determined experimentally by a “leak experiment.” In this setup, a sealing plug in electronics container was replaced by a 3D‐printed plug with a low permeability for air. The first part of the experiment was to determine the relationship between the molar air influx and the internal air pressure for a known internal volume, namely the electronics container. The second part of the experiments repeats the procedure, but in this case with the membrane module attached and submerged to avoid any significant airflow through the membranes. Using the established relationship for the molar air influx, the total internal volume was determined at *V* = 7.061 × 10^−3^ ± 0.02 × 10^−3^ m^−3^.

### CFD Model

A CFD model was developed using the OpenFOAM's solvers for transient flow and scalar transport (version 10). A fine mesh was generated from a CAD model of the membrane module, using the OpenFOAM utilities blockMesh and snappyHexmesh. The mesh was refined such that each water channel was gridded with at least 10 cells across the height *d*_1_.

The flow was simulated by a transient solver (PimpleFoam), with the inlet and outlet patches configured as constant velocity and zero gradient pressure boundary conditions, respectively. The low Reynolds turbulence model *k*‐*k*_l_‐ω^[^ ^28^, ^29^ ^]^ was selected to parameterize turbulence, as it was deemed best to cope with the transitions between turbulent flow (inlet and outlet regions) and laminar flow (between the membranes).

In a two‐step approach, first, the flow fields – forced with the flow rates of experiments I, II and III – were computed, from which 60 s averaged mean flow fields were computed. Then the averaged fields were used as flow fields for the scalar transport model (scalarTransportFoam). At the outlet patch, a zero gradient tracer (oxygen) concentration was prescribed. At the inlet patch, the tracer concentration was set to 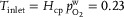 mol m^−3^.

For the membrane boundary patches, a custom boundary condition was implemented. The model for the boundary condition assumes an equilibrium between the oxygen flux through the membrane and the flux at the membrane surface interfacing with the water channel, which results in

(14)

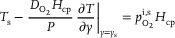

where *T*_s_ is the tracer (oxygen) concentration at the membrane surface, *y* the ordinate perpendicular to the membrane surface, and *y*_s_ the value of *y* at the membrane surface interfacing the water channel. The boundary condition model defines the oxygen concentration *T*_s_ by prescribing the partial oxygen pressure at the membrane surface interfacing the air channel, 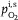. It is noted that in the analysis above, it was implicitly assumed that inside the air channels, the partial oxygen pressure at the membrane surface is equal to the average partial oxygen pressure, as would be measured by the sensors, i.e., 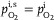. Since the diffusion coefficient of oxygen in air is much higher than that in water, the transfer rate in the air‐flow turns out to be so large that the difference between the average oxygen concentration and the oxygen concentration at the membrane surface under the conditions during the experiments are about 2 orders of magnitude smaller than the average oxygen concentration.

It is noted that the value of the membrane permeance *P* was set to *P* = 2.6 × 10^−10^ mol s^−1^ m^−2^ Pa^−1^. This value is a factor of 12.9 smaller than the value *P* = 3.39 × 10^−9^ mol m^−2^ s^−1^ Pa^−1^, as suggested in Section 2.1.

## Conflict of Interest

L.M. and P.G. are also authors of two patents: EP3819972 “Power supply system for underwater vehicles” and US11600839 “Power supply for underwater vehicles and sensors”.

## Supporting information

Supporting Information

## Data Availability

The data that support the findings of this study are openly available in [zenodo] at [https://doi.org/10.5281/zenodo.12784504], reference number [12784504].

## Appendix A Mathematical Model

To derive a mathematical model of the gill system, we consider an air flow and a water flow in rectangular channels at right angles with a membrane in between, see Figure 1b. A steady‐state situation is assumed. The mass balances for the water flow (index 1), diffusive transport through the membrane (index 2) and air‐flow (index 3) read

(A1)
$$\begin{array}{cc} \dot{n} & =Q_{1}H_{\text{cp}}\left[p_{1}\left(0\right)-p_{1}\left(l\right)\right] \end{array}$$

(A2)
$$\begin{array}{cc} \dot{n} & =Plb\left(p_{1}^{b}-p_{3}^{b}\right) \end{array}$$

(A3)
$$\begin{array}{cc} \dot{n} & =-\frac{Q_{3}}{R\Theta }\left[p_{3}\left(0\right)-p_{3}\left(l\right)\right] \end{array}$$

where  is the partial pressure of channel *i* with *i* ∈ {1, 3}, evaluated at the membrane boundary and, for the sake of simplicity, assumed constant for the whole membrane surface. The pressures *p*_*i*_ refer to profile average partial oxygen pressures. The distribution of *p*_*i*_ along the flow is given by the differential equation

(A4)
$$Q_{i}\frac{dp_{i}}{dx}+2d_{i}h_{i}\left(p_{i}-p_{i}^{b}\right)=0$$

with its solution given by

(A5)
$$p_{i}\left(x\right)=\left[p_{i}\left(0\right)-p_{i}^{b}\right]\exp\left(-\frac{2d_{i}h_{i}}{Q_{i}}x\right)+p_{i}^{b}$$

Evaluating the result for x=l and x=b, respectively, we obtain after substitution into Equation (A3)

(A6)
$$0=\dot{n}+H_{\text{cp}}Q_{1}\left[p_{1}^{b}-p_{1}\left(0\right)\right]\left[1-\exp\left(-\frac{2\mathrm{bl}}{Q_{1}}h_{1}\right)\right]$$

(A7)
$$\begin{array}{cc} 0 & =\dot{n}-Pbl\left(p_{1}^{b}-p_{3}^{b}\right) \end{array}$$

(A8)
$$\begin{array}{cc} 0 & =\dot{n}-\frac{Q_{3}}{R\Theta }\left[p_{3}^{b}-p_{3}\left(0\right)\right]\left[1-\exp\left(-\frac{2bl}{Q_{3}}h_{3}\right)\right] \end{array}$$

from which $p_{1}^{b}$, $p_{3}^{b}$, and *p*_3_(0) can be solved for, yielding

(A9)
$$p_{3}\left(0\right)=p_{1}\left(0\right)-\dot{n}\left(c_{1}+c_{2}+c_{3}\right)$$

where

(A10)
$$\begin{array}{cc} c_{1}^{-1} & =H_{\text{cp}}Q_{1}\left[1-\exp\left(-\frac{2bl}{Q_{1}}h_{1}\right)\right] \end{array}$$

(A11)
$$\begin{array}{cc} c_{2}^{-1} & =Pbl \end{array}$$

(A12)
$$\begin{array}{cc} c_{3}^{-1} & =\frac{Q_{3}}{R\Theta }\left[1-\exp\left(-\frac{2bl}{Q_{3}}h_{3}\right)\right] \end{array}$$

The coefficients *c*_*i*_ are evaluated using the values of the parameters listed in Section 2.1, with the additional parameter settings *R* = 8.31 Pa m^3^ mol^−1^ K^−1^, Θ = 293 K, and *Q*_3_ = 1.7 × 10^−7^ m^3^ s^−1^. The value of parameter *Q*_3_ corresponds to the experimental conditions and is computed from the air‐flow rate, measured at 6.6 × 10^−6^ m^3^ s^−1^, equally distributed over the 38 membrane frames. It is found that *c*_1_ and *c*_3_ can be approximated by

(A13)
$$\begin{array}{cc} c_{1}^{-1} & \approx 2H_{\text{cp}}\;b\;l\;h_{1} \end{array}$$

(A14)
$$\begin{array}{cc} c_{3}^{-1} & \approx \frac{Q_{3}}{R\Theta } \end{array}$$

so that $c_{1}^{-1}=2.5\times 10^{-12}$ mol s^−1^ Pa^−1^, $c_{2}^{-1}=1.4\times 10^{-10}$ mol s^−1^ Pa^−1^, and $c_{3}^{-1}=7.1\times 10^{-11}\;\;\text{mol}$ s^−1^ Pa^−1^. Their ratios evaluate to *c*_1_/*c*_2_ = 56, and *c*_1_/*c*_3_ = 28.

## Appendix B Scaling Analysis

We apply the Pi‐theorem^[^ ^27^ ^]^ to the problem of oxygen transfer from the water phase into the air phase inside the membrane module. This membrane module contains *N* air channels, each with height *d*_3_, and effectively also *N* water channels, each with a height *d*_1_. The water and air channels are separated by a membranes with dimensions *l* and *b*. For oxygen to be transported from the water flow into the air‐flow, a partial oxygen pressure gradient Δ*p* needs to exist across the membrane. As a result, the partial oxygen pressure at the membrane boundary in the water channel will be slightly lower than in the core of the water flow and on the boundary in the air channel slightly higher than in the core of the air‐flow. Therefore, the oxygen permeate flux will depend on *i*) how easily oxygen can be transported from the core of the water flow to the membrane, *ii*) how permeable the membrane is to oxygen molecules, and *iii*) how easily oxygen can be transported from the membrane boundary on the air channel's side into the core air flow. For the reasons voiced in Appendix A, we will assume that the latter effect is negligible.

We proceed by listing all parameters we think may affect the oxygen permeate flux. These are, in no particular order, Henry's coefficient of solubility of oxygen in water, *H*_cp_, the kinematic viscosity of water, ν, the flow speed of the water through the channels, *u*, the height of the water channel *d*_1_, the pressure gradient Δ*p*, the coefficient of molecular diffusion of oxygen in water , the permeance of the membrane (i.e., membrane permeability divided by membrane thickness), *P*, and the dimensions of the membrane *l* and *b*. A total of nine dimensional parameters are identified that govern the behavior of the membrane module:

(B1)

A total number of four dimensions are involved (in SI‐units kg, mol, m and s), so that we also seek four independent dimensional parameters, i.e., parameters which dimensions cannot be expressed by any combination of the other independent dimensional parameters. According to the Pi‐theorem, the non‐dimensional oxygen permeate flux, Π can be formulated as a function Φ of five non‐dimensional parameters Π_*i*_:

(B2)
$$\Pi =\Phi (\Pi_{1},\Pi_{2},\Pi_{3},\Pi_{4},\Pi_{5})$$

Choosing Δ*p*, *H*_cp_, ν and *d*_1_ as independent parameters, we find

(B3)

If the scaling analysis is applicable, then Equation (B2) should be an abstraction of the non‐dimensional form of the mathematical model in Equation (7), which, after substituting from Equations (8) and (9), can be written as

(B4)
$$\frac{\dot{n}}{\Delta pH_{\text{cp}}\nu d_{1}}=\frac{b}{d_{1}}\;\frac{l}{d_{1}}\;{\left(\text{Sc}\;{\text{Sh}}^{-1}+\frac{H_{\text{cp}}\nu }{Pd_{1}}\right)}^{-1}$$

Clearly, the left‐hand sides match, and from substitution, it follows that the function Φ reads

(B5)
$$\Phi =\Phi_{1}\left(\frac{\Pi_{4}\;\Pi_{5}}{\Pi_{3}\;{\text{Sh}}^{-1}+\Pi_{2}^{-1}}\right)$$

A new (unknown) function Φ_1_ is introduced to account for the fact that the mathematical model considers one channel with a well‐defined flow, and the scaling analysis considers the whole membrane module, with multiple channels and inhomogeneous flow. For simplicity, it could be assumed that Φ_1_(*x*) = *Nx*, but the exact form does not matter for our argument. Key is the observation that Sherwood number is still present in argument to Φ_1_ in Equation (B5), but Π_1_ is not. This can only be reconciled if and only if Sh is a function of (at least) Re.

For the sake of completeness, it is noted that if the oxygen transfer due to the air channel were to be taken into account, a further independent parameter would be introduced (Θ), as well as three additional non‐dimensional parameters. The expression for Φ_1_ would become more elaborate. However, the conclusion on the relationship between Sh and Re would not change.
