# Supplementary material for: A Fuel Cell Power Supply System Equipped with Artificial Gill Membranes for Underwater Applications
Source: Adv Sci (Weinh). 2025 Jan 10;12(11):2410358. doi: 10.1002/advs.202410358 (PMC11923934; doi:10.1002/advs.202410358)
Supplement: Supplementary file 1 — Supporting Information [file ADVS-12-2410358-s001.pdf]

ADVANCED SCIENCE
Open Access

Supporting Information

for *Adv. Sci.*, DOI 10.1002/advs.202410358

# A Fuel Cell Power Supply System Equipped with Artificial Gill Membranes for Underwater Applications

*Lucas Merckelbach* and Prokopios Georgopanos**

# Supporting Information
to
# A Fuel Cell Power Supply System Equipped with Artificial Gill Membranes for Underwater Applications

https://doi.org/10.1002/advs.202410358

*Lucas Merckelbach*[1] *Prokopios Georgopanos*[2]

[1]Helmholtz-Zentrum Hereon, Institute of Coastal Ocean Dynamics, Max Planck Str. 1, 21502, Geesthacht, Germany
Email: lucas.merckelbach@hereon.de
[2] Helmholtz-Zentrum Hereon, Institute of Membrane Research, Max Planck Str. 1, 21502, Geesthacht, Germany
Email: prokopios.georgopanos@hereon.de

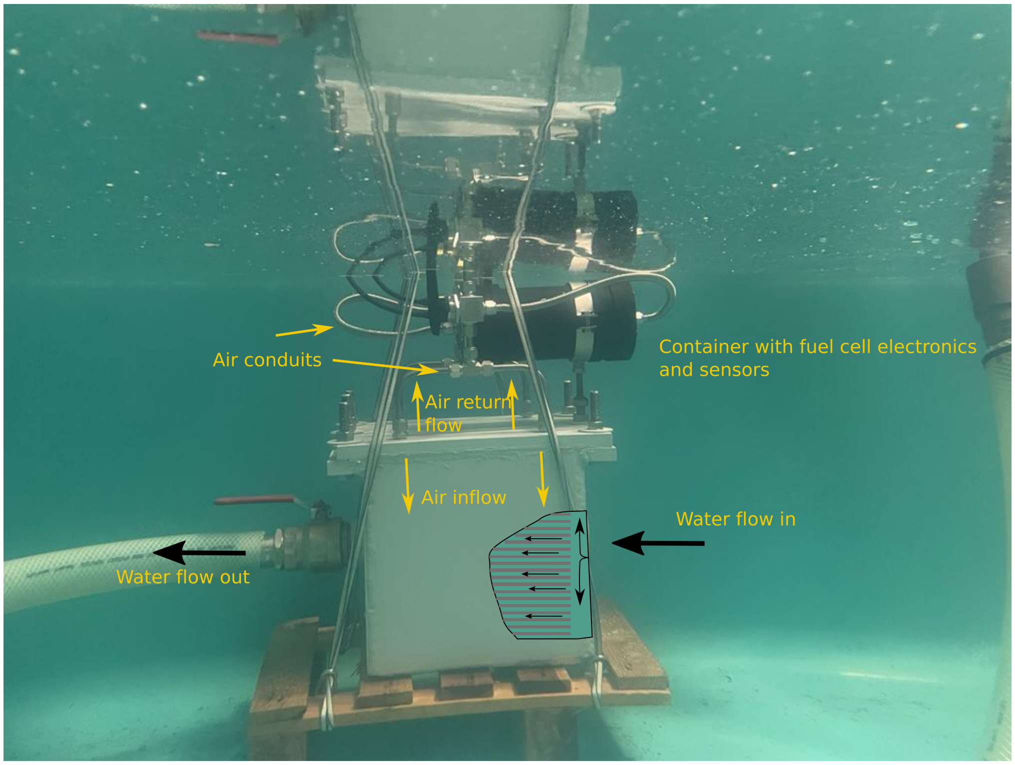


Figure S1: Annotated image of the prototype operating underwater. The black container houses the fuel cell, sensors and all electronics, see Figure 2c of the manuscript, and it is connected to the gray membrane module by two air conduits (one inlet and one outlet). The membrane module has a water inlet and outlet and a stack of membranes; see Figure 2 of the manuscript. A water pump (not in the picture), connected to the outlet tube, forces the water through the membrane module. Two cables to the left of the black electronics container (cutting the water surface) are connected to a computer for data logging and provide the circuitry for an adjustable electrical load of the fuel cell.

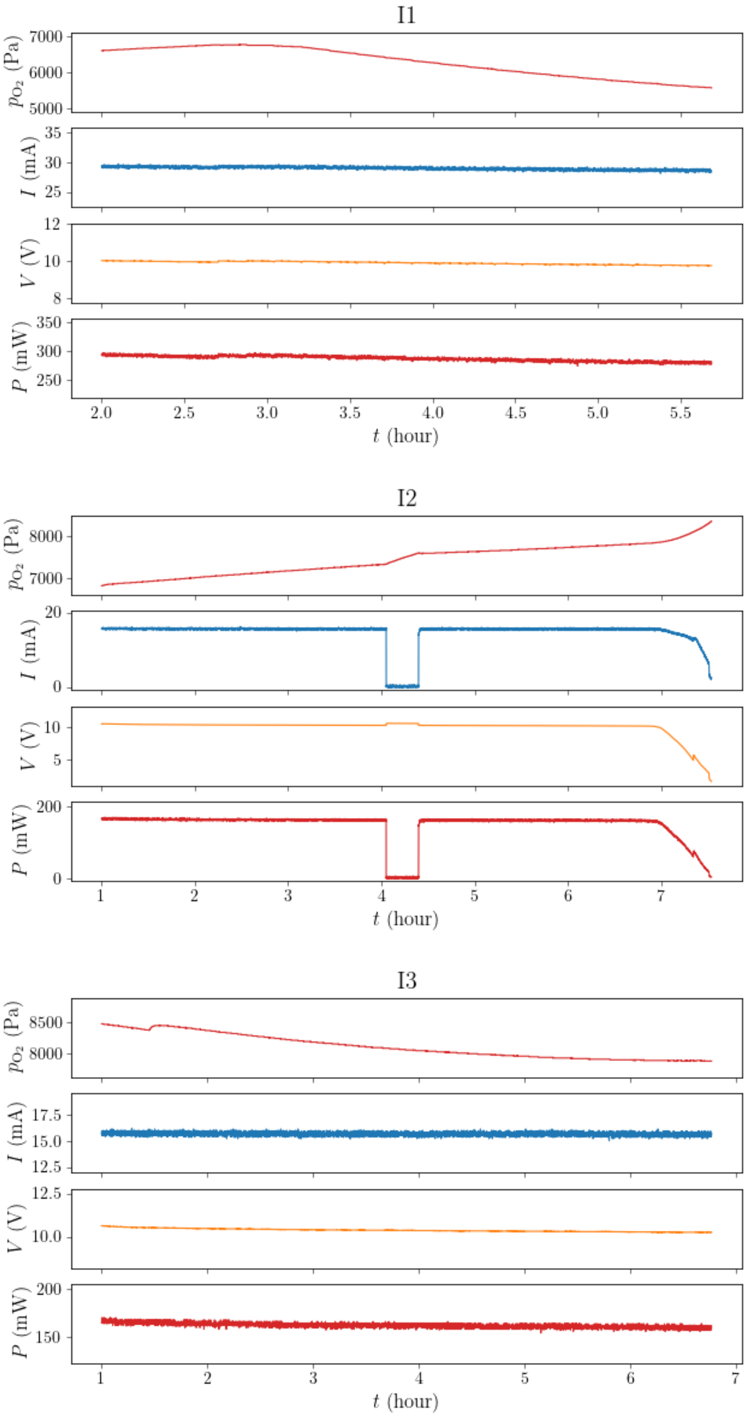


Figure S2: Raw measurement data for the internal partial oxygen pressure, as well as measured data on the electrical current, voltage and power generated by the fuel cell for experiment I, all three replicates.

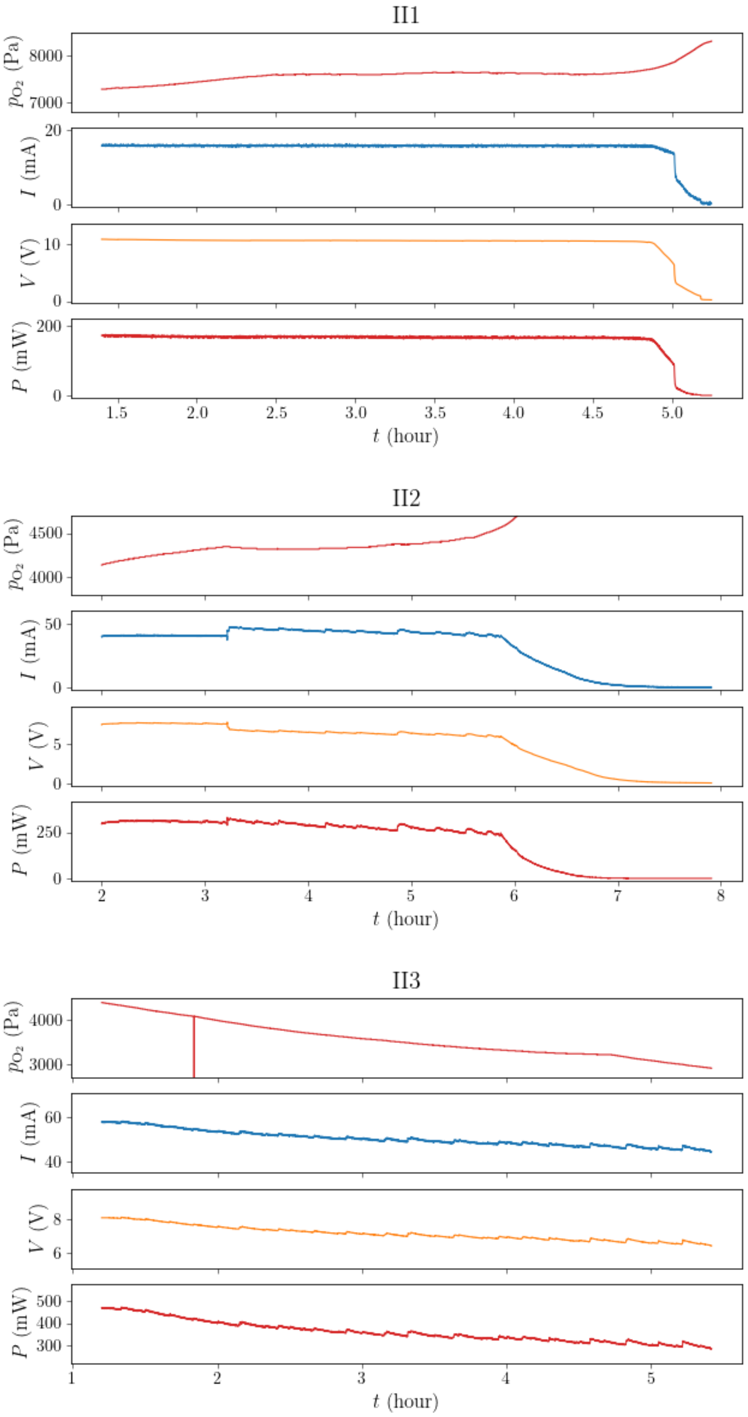


Figure S3: Raw measurement data for the internal partial oxygen pressure, as well as measured data on the electrical current, voltage and power generated by the fuel cell for experiment II, all three replicates.

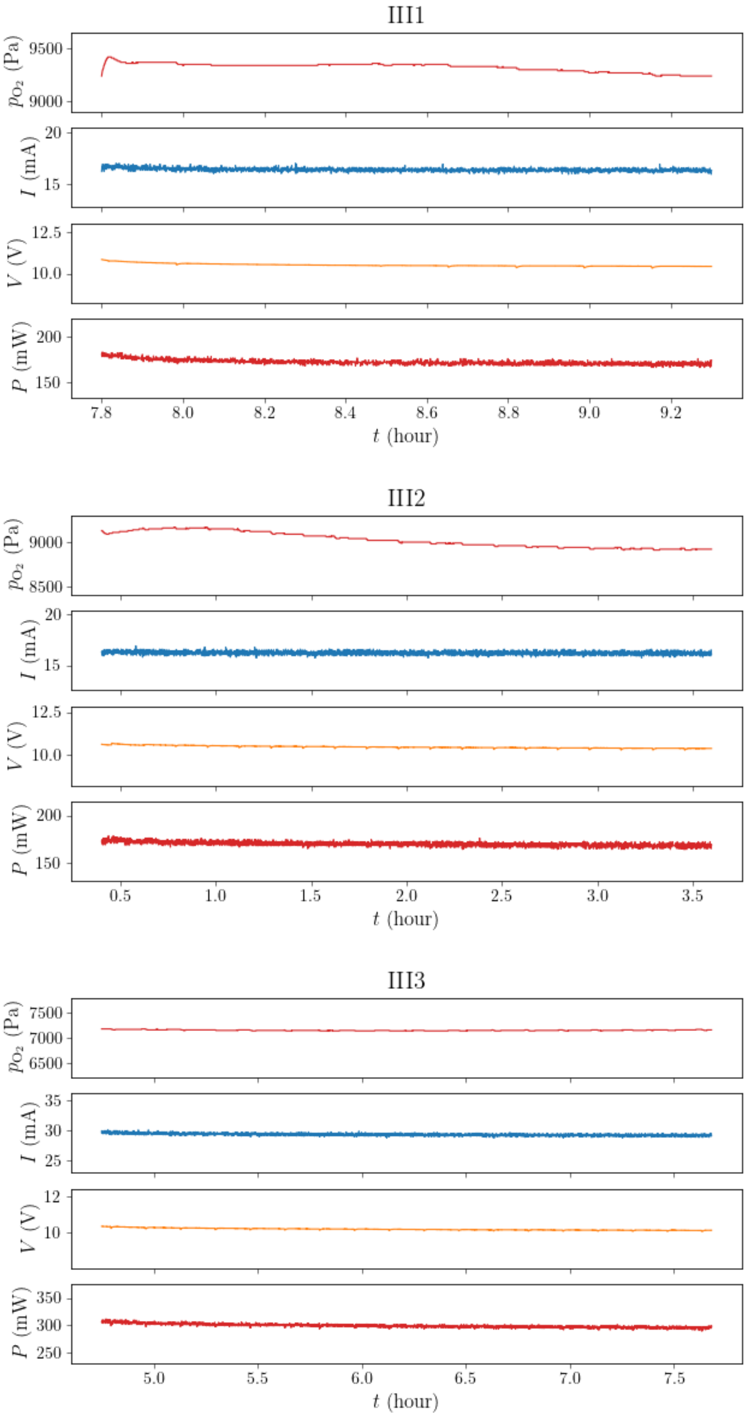


Figure S4: Raw measurement data for the internal partial oxygen pressure, as well as measured data on the electrical current, voltage and power generated by the fuel cell for experiment III, all three replicates.
